# Identifying Palaeolithic birch tar production techniques: challenges from an experimental biomolecular approach

**DOI:** 10.1038/s41598-023-41898-5

**Published:** 2023-09-07

**Authors:** Paul R. B. Kozowyk, Liliana I. Baron, Geeske H. J. Langejans

**Affiliations:** 1 Department of Materials Science and Engineering, Delft University of Technology, Delft, 2628CD The Netherlands; 2 Department of Chemical Engineering, Delft University of Technology, Delft, 2629HZ The Netherlands; 3 Palaeo-Research Institute, University of Johannesburg, Johannesburg, 2092 Gauteng South Africa

**Keywords:** Archaeology, Gas chromatography, Mass spectrometry

## Abstract

The intentional production of birch bark tar by European Neanderthals as early as 190,000 years ago plays an important role in discussions about the technological and behavioural complexity of Pleistocene hominins. However, research is hampered because it is currently unknown how Neanderthals were producing birch tar. There are several different techniques that could have been employed, but these differ in their apparent production complexity, time and resource efficiency. Identifying production processes in the archaeological record is therefore paramount for furthering research on the technical behavioural repertoire. Organic biomarkers, identified with Gas Chromatograph–Mass Spectrometry (GC–MS), have been used to identify possible production processes during the Neolithic. Here we test whether these biomarkers can also distinguish Palaeolithic (aceramic) tar production methods. We produced tar using five different methods and analysed their biomolecular composition with GC–MS. Our results show that the biomarkers used to distinguish Neolithic tar production strategies using ceramic technology cannot be reliably used to identify tar production processes using aceramic Palaeolithic techniques. More experimentation is required to produce a larger reference library of different tars for future comparisons. To achieve this, complete GC–MS datasets must also be made publicly available, as we have done with our data.

## Introduction

Archaeological evidence of adhesive production and use by Neanderthals and early modern humans has played a key role in discussions about the evolution of technological and behavioural complexity among these species^1–7^. Depending on the adhesive material and the production method employed in the past, the use of adhesives required different degrees of planning, in depth knowledge of material properties and their interactions, abstract thinking, and significant working memory capabilities^1,3,8–12^.

Birch bark tar is currently the oldest known adhesive substance, dating to at least 190,000 years ago (kya) at Campitello Quarry, Italy^13^. It can therefore be unambiguously attributed to European Neanderthals. Other notable archaeological finds come from Königsaue, Germany (> 43 kya)^14^ and Zandmotor, The Netherlands (~ 50 kya)^4^. All finds are similar lumps of tar encasing (or once having encased) a flint object and can also be attributed to Neanderthals. Unlike resins and gums that are exuded from trees in a naturally sticky state, birch bark tar must be intentionally manufactured. There are a number of different ways that this can be done that vary in complexity, but all methods require some degree of knowledge about the use of fire to transform white bark into sticky black tar^12,15^. Despite the initial investment in birch tar manufacture, rather than collecting a naturally sticky resin, it was likely intentionally selected for because it has a number of material properties that are favourable for a stone tool hafting adhesive^16^. Birch bark tar has also been found in Neolithic, Bronze Age, Iron Age, Roman and Medieval contexts, and geographically spans from Europe to central China^17–23^. Discerning production processes can therefore shed light on the technological capabilities and material choices of ancient peoples across a wide temporal and geographic range.

There have been a number of experimental studies exploring prehistoric methods of birch tar production^24–27^. There are least four different ways of producing usable quantities of birch tar using technology and materials available to Neanderthals^1,2^. These vary in time, yield efficiency, and apparent production complexity^1,28^. The known variation between tar production processes has resulted in disagreement about the technological and behavioural capabilities of Neanderthals^2,15,29^. Methods that are seemingly more complex have been used to argue that Neanderthals used complex technologies to mitigate ecological risk at the northern edge of their habitat. The discovery of production methods requiring fewer resources and production steps suggests that birch tar should not be used as a proxy for Neanderthal complexity^2^ (but see also^15,29^). It was previously unknown which production methods were actually being used in the Middle Palaeolithic. However, it has recently been suggested that the tar lump found at Königsaue was made using one of two methods involving below-ground tar production^30^. Further distinguishing between these two methods was not possible. It is also currently unknown whether the same techniques were used to make the tar lumps found at Campitello, Italy, and Zandmotor, The Netherlands. In order to systematically progress the discussion about Neanderthal tar production and, by proxy, behaviour and cognition, it is therefore necessary to identify and more accurately distinguish ancient tar production processes.

Identifying tar production processes can be carried out by two different pathways. (1) Finding direct evidence of production structures or objects. (2) by identifying biomarkers within the tar that are only produced under circumstances arising from specific production processes. The first approach would include finding cobbles or stones used in the production with birch bark tar residues and use-wear traces on tools from preparing the raw materials. Small pit features and baked earth containing traces of tar could be used to indicate other methods of production^31^. Finding such traces from the Palaeolithic is problematic due to the taphonomy of organic residues^32^, and the size and equifinality of production processes^1,33^ (e.g., compare Huisman^34^ and Crombé^35^). Tar production may also have been done outside of camps, near the raw material sources, or at opportunistic times, where archaeologists are unlikely to easily discover or recognise them.

The second, and more promising approach to identifying production techniques, requires the study of specific biomarkers using chemical/spectrographic techniques such as gas chromatography-mass spectrometry (GC–MS). Using GC–MS, raw material sources can be identified by the presence or absence of specific chemical compounds, referred to as biomarkers. These act as a chemical fingerprint that are unique to specific organic materials^36^. For example, tar is a complex blend of aromatic hydrocarbons^7^, but has different terpenes and fatty acids depending on the raw material source^37^, making initial distinctions between raw material sources such as pine and birch bark relatively straightforward^38^. Identifying production processes takes this technique one step further. Key biomarkers are altered in specific ways depending on the different physical, chemical, and biological forces that act on them^39,40^. These changes to biomarkers can result from natural processes, but they can also result from anthropogenic forces, such as thermal degradation due to heating temperatures, oxygen abundance, or chemical interactions during manufacture. These types of alterations therefore the potential to provide direct insight to the original production and manipulation of the material by those who made and used it.

Research has already been conducted studying effects of heating conditions on a range of birch tar biomarkers using Neolithic production with ceramic containers^37^. Ceramic tar production falls into two categories: (1) single pot, and (2) double pot production. In single pot production, the tar is collected inside the same chamber as the reaction material, so it can be exposed to prolonged high temperatures in a low oxygen environment. In double pot production, the tar drips into a separate, cooler, chamber below the reaction material. These processes differentially effect the chemical composition of the tar, which could be determined by comparing different biomarkers^37^. However, it is unknown how aceramic production processes will affect these biomarkers or whether the same methodology can be used to differentiate between Palaeolithic techniques. Here we produced birch tar using four possible Palaeolithic methods and one laboratory technique and analysed the chemical composition with GC–MS to compare the biomarkers with those used to describe ceramic tar production. We then assess the suitability of these biomarkers and other compounds for identifying Palaeolithic birch tar production techniques.

## Materials and methods

### Experimentally produced tars

**Figure 1:**
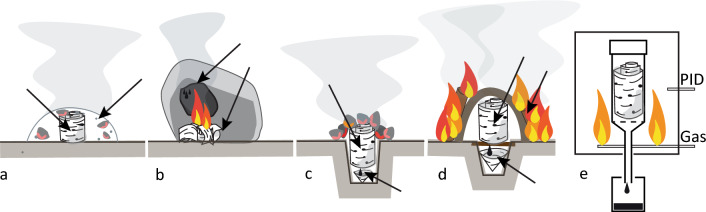
Tar production method illustrations. Five methods used to produce tar include: (**a**) ash mound (**b**) condensation, (**c**) pit roll, (**d**) raised structure, and (**e**) laboratory furnace. Black arrows indicate the positions of thermocouples used to record temperatures during production. In the laboratory furnace, temperature was controlled with a PID device in the centre of the chamber, which regulates the gas flow to maintain a relatively constant temperature.

To measure the biomarkers of Palaeolithic birch bark tar we produced tar using five distinct processes (Fig. 1). Four methods could have been employed by Neanderthals using materials and technology available to them. They are referred to as the (1) ash mound, (2) condensation, (3) pit roll, and (4) raised structure. (5) lab produced tar, was done in a laboratory kiln and subject to a three year field weathering experiment to assess environmental degradation of controlled tar^32^. The production processes for each method are described as follows:

1. Ash mound—A small roll of birch bark is buried in a pile of hot ashes and embers. After 30–40 min the roll is excavated and as it is opened hot tar can be scraped from between the layers of bark^1^. This method most closely resembles a ceramic single pot production. The roll of bark and pile of ash excludes oxygen to the centre, and heat is provided from the surrounding material. One ash mound sample was taken for analysis (AM1).
2. Condensation—Small amounts of birch bark are burned next to a near vertical stone surface. The smoke condenses on the stone surface and can be scraped off periodically with a flint flake and collected into a small ball of tar^2^. This method is the least like any ceramic tar production processes as the tar is produced entirely in an open-air environment and not in a container or reduced oxygen environment. Two condensation experiments were conducted and one sample was taken from each for analysis (C1, C2).
3. Pit roll—A pit approximately 10 cm deep by 7 cm in diameter is dug and a small birch bark cup is placed at the bottom. A tightly rolled birch bark roll is placed into the hole, sticking up a few centimetres above the ground, and hot embers are placed over and around the roll of bark. This method lies somewhere between a single and double pot process, as there is no distinct separation between the bark roll and the cup in the bottom of the pit. However, the bottom of the pit remains cooler than the top, and as tar forms it drips into the pit, as in a *per descensum* or double pot method^1^. Tar can also be collected from inside the remaining roll of bark. Two pit roll experiments were conducted, and one sample was taken from each for analysis (PR1, PR2).
4. Raised structure—A pit approximately 10 cm by 10 cm is dug and a birch bark cup is placed at the bottom. A screen or mesh of twigs is placed over the pit. On top of this mesh round pebbles may be placed, and then the roll of birch bark on top of the pebbles. The entire system is then covered in a mud dome-like structure and a fire is lit all around for several hours. This is the Palaeolithic equivalent of a double pot method^1^. Two raised structure experiments were conducted (RS1, RS2). After approximately 325 min, the fire stopped being fed. RS1 was left to cool gradually before opening and RS2 was opened almost immediately after. One sample from each was taken for analysis.
5. Lab tar—Tar was produced in a double pot process using a PID (proportional–integral–derivative) controlled muffle furnace. Birch bark was placed in a stainless steel work tube containing a perforated base and a pipe leading to a glass jar outside of the furnace to collect the tar. The bark was heated for approximately 2.5 h at 350–405 °C. After the tar was produced, it was boiled over an electric hot plate until the consistency was solid at room temperature. At this point 43% by mass of the original tar produced was remaining. It was then used to haft a flint flake in a wooden cleft handle and left to weather at the Horsterwold Experimental Outdoor Research Facility, Leiden University, for three years^32^. One lab tar sample was taken for analysis (L1).

Tars for Am1, PR2, RS2, and Lab1 were sampled from previously published experiments^1,32^. Tars for C1, C2, PR1, and RS1 samples were produced specifically for this study using the same bark, and identical experimental setups as PR2 and RS2. Thermocouples were placed in or near the production assemblies to record the temperatures each process exposed the tar and bark to (Fig. 1). However, for methods such as the condensation technique, the temperatures are highly variable, as it depends on whether the thermocouple probe was directly in or above the flame produced by the bark, which was constantly shifting. Ambient temperature during the production of AM1, PR2, and RS2 varied between approximately 1 and 11 °C over the course of the experiments. Ambient temperatures during the production of C1, C2, PR1 and RS1 varied between approximately 5 to 12 °C. Experiments were conducted in relatively sheltered area to reduce the influence of wind.

### Analytical methods

To study the biomolecular composition of each experimental sample we used the GC–MS protocol outlined by Rageot and colleagues^37,41^. Samples were ground and then 1 mg of each the sample was extracted with 1 mL of dichloromethane (DCM) HPLC grade purchased from TCI. Extraction was done by ultrasonication for 30 min. An aliquot of the supernatant was evaporated until dryness under a gentle stream of nitrogen. Derivatization was made using bis(trimethysilyl)trifluoroacetamide (BSTFA) containing 1% trimethylchlorosilane purchased from Sigma–Aldrich. Ten microlitres of DCM, 5 µL of pyridine (purchased from TCI) and 50 µL of BSTFA were added to the dry extract and the reaction took place for 20 min at room temperature. It was then evaporated to dryness under nitrogen stream at 30 °C and recovered with DCM. One microlitre of the sample was then injected into GC–MS.

The GC–MS analyses were performed on an Agilent 7890B gas chromatograph system with a split/splitless inlet, coupled with an Agilent 5977B EI MSD interface, an FID and a splitter with corresponding EPC pressure control to achieve this. The GC was fitted with a nonpolar Agilent J&W DB5 MS column (30 m × 0.25 mm i.d.; 0.25 μm film thickness). The samples were introduced in a split/splitless injector used in the splitless mode at 300 °C with septum purge flow 3 ml/min. The oven temperature was held isothermally for 2 min at 50 °C, ramped at 10 °C per minute to 150 °C, ramped at 4 °C per minute to 320 °C, and held at that temperature for 20 min. The analysis was carried out using helium as carrier gas at a constant flow rate of 1.6 mL/min (average velocity 32.146 cm/sec). The temperature of FID was set at 340 °C and hydrogen flow was 30 ml/min, synthetic air 400 ml/min, and nitrogen 30 ml/min. The temperatures of the ion source were set at 230 °C and the transfer line at 280 °C. The mass spectrometer was monitored to scan 35–950 m/z with an ionizing voltage at 70 eV. Total run time was 75 min and solvent delay was 4 min. GC–MS chromatogram was interpreted using National Institute standard and Technology (NIST). The mass spectra were matched against those of authentic standards (betulin and lupeol), by using data previously published^37^ and the NIST library^42^.

To further evaluate the differences between production processes we conducted principal component analysis (PCA) based on the relative abundance from peak area percentage of the biomarkers listed in Table 1. PCA was done using ClustVis (BETA)^43^. Original values are ln(x + 1)-transformed. Unit variance scaling is applied to rows; SVD with imputation is used to calculate principal components. All raw GC–MS and PCA is publicly available at the 4TU.Centre for Research Data^44^.

**Table 1:** Percentage of peak area and retention times (RT) of fatty acids and biomarkers present in lab produced (L1), ash mound (AM1), condensation (C1, C2), pit roll (PR1, PR2), and raised structure (RS1, RS2) tars.

Compound	RT	L1	AM1	C1	C2	RS1	RS2	PR1	PR2
Guaiacol-TMS	11.1	0.00	0.00	0.00	0.00	0.00	0.00	0.02	0.00
Glycerol, 3TMS derivative	11.7	0.22	0.00	0.26	0.30	0.00	0.00	0.12	0.00
Catechol, 2TMS derivative	12.4	0.00	0.00	0.26	0.84	0.00	0.00	0.42	0.00
Levoglucosan, 3TMS derivative	19.3	0.00	0.00	0.69	1.44	0.00	0.00	0.16	0.00
Tetramethylnaphthalene	20.2	0.00	0.00	0.00	0.00	0.86	0.00	0.09	0.00
Pentadecanoic acid, TMS derivative	24.8	0.00	0.00	0.00	0.00	0.38	0.00	0.07	0.00
Palmitic acid TMS derivative	27.0	0.50	0.02	0.57	0.66	1.77	0.23	0.62	0.00
Bisphenol A, 2TMS derivative	27.3	6.54	0.00	2.95	2.30	1.29	0.00	0.83	0.00
Heptadecanoic acid, TMS derivative	29.0	0.00	0.00	0.00	0.00	0.50	0.00	0.06	0.00
9,12-Octadecadienoic acid (Z,Z)-, TMS derivative	30.0	0.00	0.00	1.30	0.98	2.06	0.25	0.28	0.26
9-Octadecenoic acid, (E)-, TMS derivative/13-Octadecenoic acid, (E)-, TMS derivative	31.1	0.00	0.00	0.00	0.00	0.50	0.00	0.08	0.00
Stearic acid, TMS derivative	31.3	0.14	0.01	0.19	0.13	1.10	0.22	1.15	0.00
cis-13-Eicosenoic acid	33.0	0.00	0.00	0.43	0.00	5.37	1.48	0.73	0.00
10-Nonadecenoic acid, (Z)-, TMS derivative	34.6	0.00	0.00	3.21	1.92	0.00	0.00	0.16	0.00
11-Eicosenoic acid, (E)-,TMS derivative	35.1	0.00	0.00	1.02	0.77	0.00	0.00	0.00	0.16
Erucic acid	36.8	0.00	0.15	0.71	0.43	4.45	2.22	0.96	0.00
Heneicosanoic acid, TMS derivative	37.2	0.00	0.00	0.55	0.39	0.00	0.00	0.00	0.18
13-Docosenoic acid, (Z)-, TMS derivative	38.9	0.00	0.00	2.96	2.27	0.00	0.17	0.06	0.33
Behenic acid, TMS derivative	39.0	0.13	0.00	1.02	0.95	0.23	0.21	0.02	0.16
10,12-Docosadiynedioic acid, 2TMS derivative	41.0	0.00	0.00	0.71	0.31	0.00	0.00	0.00	0.00
lupadiene	46.0	23.17	6.78	1.84	1.53	0.50	11.53	1.95	6.10
α-Betulin I	46.4	0.00	1.95	6.46	6.84	7.54	0.00	5.65	1.04
Lupadienol, TMS derivative	47.6	8.71	1.21	0.00	0.00	0.50	2.57	1.47	2.78
	48.6	5.79	7.63	14.74	14.68	0.50	1.90	6.34	4.33
Allobetul-2-ene	49.5	48.70	2.04	1.21	1.00	12.17	3.22	1.32	1.33
Lupenone	50.6	0.00	2.67	0.00	0.00	0.00	1.42	0.00	0.65
Lupeol trimethylsilyl ether	51.0	0.00	23.50	0.44	3.83	5.21	23.91	4.51	24.63
Betulone, TMS derivative	52.8	0.00	1.50	1.81	1.63	0.50	0.00	0.96	0.00
Betulin, bisTMS	53.2	0.00	44.52	53.67	54.34	31.52	49.76	66.79	56.03
Betulinic acid, O,O-bis-TMS	53.5	0.00	7.45	2.97	2.45	0.11	0.91	3.91	2.02
3-Oxoallobetulane	53.9	6.10	0.57	0.00	0.00	2.27	0.00	0.44	0.00
Oleanolic acid 2TMS	54.5	0.00	0.00	0.00	0.00	0.00	0.00	0.81	0.00
β-Amyrin	NA	0.00	0.00	0.00	0.00	0.00	0.00	0.00	0.00

Lupadienol was identified by peaks with retention times at both 47.6 and 48.6.

## Results

### Production temperatures

**Figure 2:**
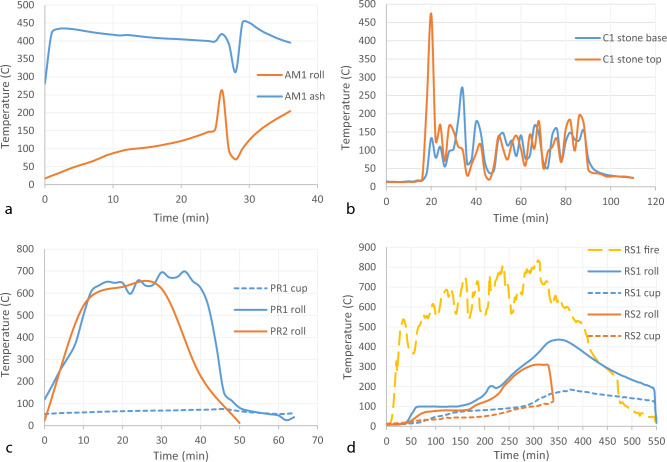
Temperature curves of the four Palaeolithic methods. Plots show (**a**) Ash mound, (**b**) condensation, (**c**) pit roll, (**d**) raised structure. Recorded temperatures for the condensation method are highly variable because the flaming bark and stone was being moved regularly as new bark was added and the stone was moved in order to scrape tar. Temperatures in the flame were likely much higher than what is recorded here.

Temperatures reached during production affect the chemical composition of the final tar. Prolonged heating will likely lead to more thermal degradation of the original birch bark constituents. We therefore recorded the temperatures reached for each production technique. Temperatures for the condensation process were highly variable, so they cannot be used as a reliable indication that this was the temperature the tar was produced at or exposed to. They were therefore only recorded for one experiment (C1). The two pit roll production experiments reached similar temperatures inside the bark as the roll was burned. The two raised structures were heated at a similar rate but RS1 reached a higher final temperature inside the bark roll and was left to cool gradually while RS2 was opened and cooled more quickly (Fig. 2). Temperature for AM1 peaked and cooled at around 26 min when the roll was opened to check for the formation of tar and the re-buried in ash to complete the process.

### Biomarkers

Birch bark is composed of approximately 25–34% betulin (Lup-20(29)-ene-3β,28-diol), and contains other triterpenes with lupane skeletons^45,46^. Birch bark tar, therefore, usually contains betulin, lupeol, and other thermally altered degradation products of these compounds such as lupenone (lup-20(29)-en-3-one), lupadienol (lup-2,20(29)-dien-28-ol), betulone (lup-20(20)-en-3-one-28-ol), and lupadiene (lup-2,20(29)-diene)^37^. Betulin, lupeol and their degradation products are the chemical biomarkers most commonly used to identify birch bark tar in the archaeological record^4,14,21^. Among tars produced with ceramics, behenic acid (docosanoic acid), betulinic acid ((3β)-3-Hydroxy-lup-20(29)-en-28-oic acid), and other fatty acids and diacids are found only in double pot tars and not in single pot tars^37^. These fatty acids should show comparable patterns with tar produced in similar ways without ceramic pots. For example, the raised structure most closely resembles the ceramic double-pot process, and the ash mound most closely resembles a single-pot process. Behenic and betulinic acid should therefore be present in the raised structure and absent in the ash mound tars. Degradation products of betulin and lupeol should also be present in tars subject to open air heating, such as the condensation method, or the lab tar that was left to weather naturally for three years.

**Figure 3:**
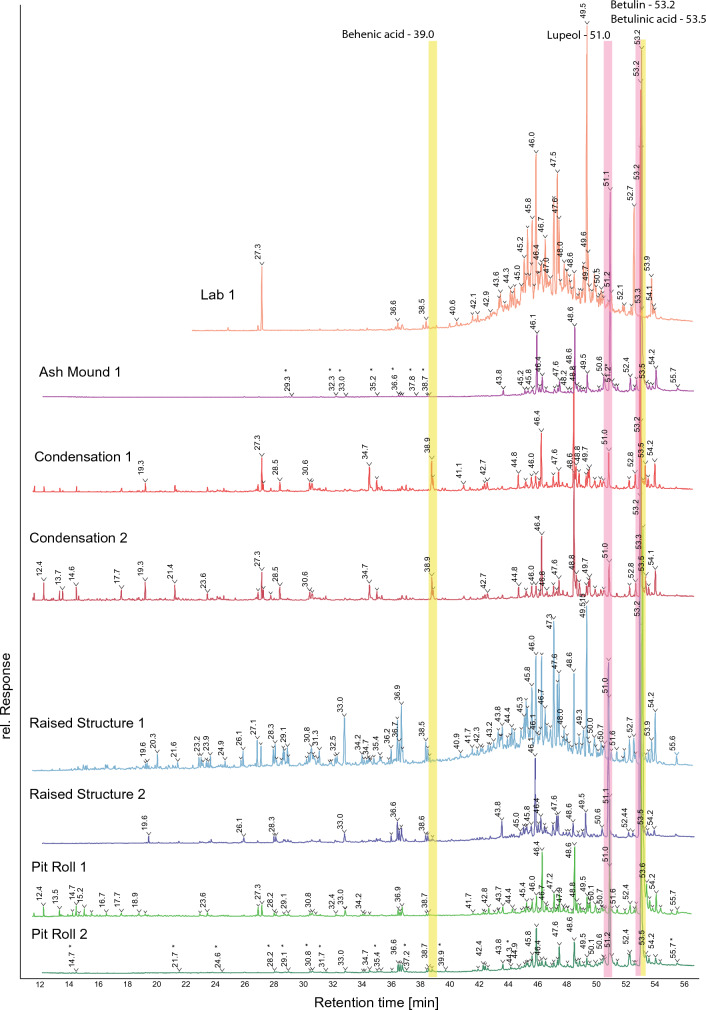
Chromatograms of AM1, C1, C2, PR1, PR2, RS1, RS2. Intra- and inter-method variation is high between methods. Highlighted regions show behenic and betulinic acid (yellow), which vary between ceramic production processes, and betulin and lupeol (pink), the two main constituents of birch bark, and the primary biomarkers used to identify archaeological birch bark tars.

**Figure 4:**
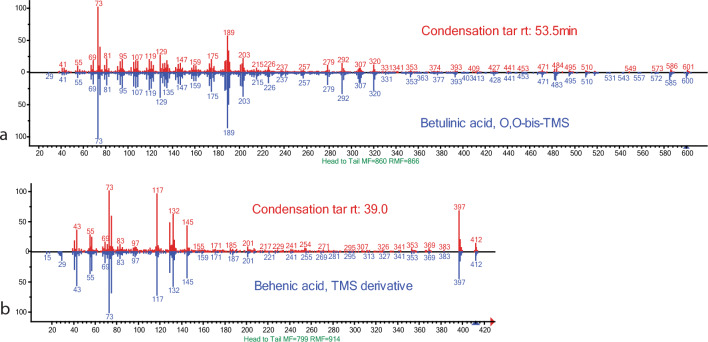
Mass spectra comparing experimental biomarkers with pure reference material. Betulinic acid (**a**) and behenic acid (**b**) from condensation tar and pure reference sample.

Behenic acid is absent in the ash mound tar, and present in all others (Table 1; Fig. 3). This aligns with expectations derived from ceramic tar production^37^. However, both tar samples made with condensation also contain behenic acid despite it being considerably different to double-pot or raised structure methods. Identified at a retention time of approximately 39.0 min in C1 and C2, the peaks are a clear match with a pure behenic acid reference^42^ (Fig. 4). Other fatty acids said to distinguish single and double pot tars are present in no clear pattern among our different tars. They are primarily absent in the ash mound tar and the lab produced tar. Some fatty acids are also present in only one of the two samples for both the pit roll and raised structure tars (Table 1).

We found betulinic acid in tar from four out of five of our production methods (Fig. 4). Notably, it is present in the ash mound tar, which most closely resembles a single pot process. Betulinic acid is also present in tar produced through condensation, in the pit roll, and in the raised structure (Table 1), so it cannot be used to distinguish between these methods. It is absent in the lab produced degradation experiment. Betulinic acid can be destroyed by natural decay or open-air heating^37^, so its absence in the degraded sample is expected. However, the condensation process also subjects the tar to open air heating during its production, so its presence in C1 and C2 is surprising.

Another interesting difference is the regular presence of betulone in all ceramic tars^37^ and its absence in L1, RS2, and PR2. Betulone is formed during the natural or thermal decay (oxidation) of betulin, and along with lupenone, lupadienol and lupadiene, is considered a useful biomarker for the identification of archaeological birch bark tar. Similarly, lupenone was absent in four of our experimental Palaeolithic tars (L1, C1, C2, RS1, PR1; Table 1). Lupadienol, and lupadiene, were present in all of our samples.

In addition to the biomarkers used to differentiate ceramic production processes, we also included phenolic compounds, naphthalenes, an anhydrous sugar, and a 14-carbon saturated fatty acid in our results (Table 1) to further help explain the differences between production processes. The phenolic compound guaicol (2-methoxyphenol) is present in PR1. Catechol (benzene-1,2-diol) and levoglucosan ((1 R ,2 S ,3 S ,4 R ,5 R )-6,8-dioxabicyclo[3.2.1]octane-2,3,4-triol) are present in C1, C2 and PR1. Levoglucosan is formed during the pyrolysis of starch and cellulose^47^, suggesting localized pyrolysis conditions are present in the condensation method, despite it being a fully open-air process. 1,2,3,4-Tetramethylnaphthalene and pentadecanoic acid are both present only in RS1 and PR1.

**Figure 5:**
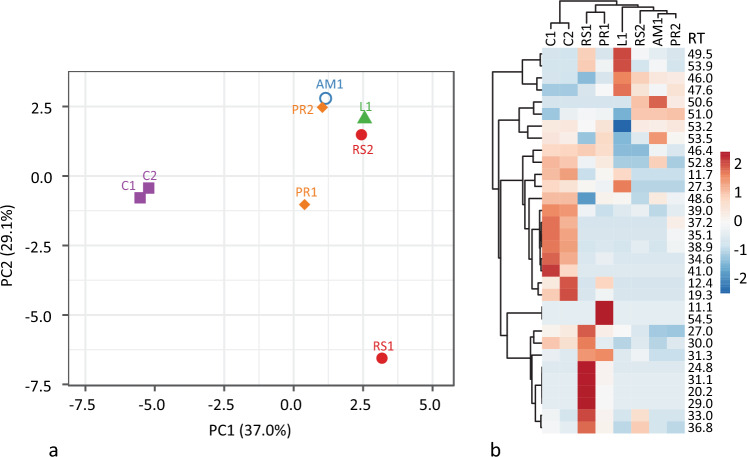
PCA of experimental tar samples (**a**) and heat-map of peak area % (**b**). X and Y axis show principal component 1 and principal component 2 that explain 37.9% and 29.1% of the total variance, respectively. N = 8 data points. Condensation tars (C1, C2) show the least intra-method biomarker variation and raised structure (RS1, RS2) show the most variation. Lab produced tar is more similar to AM1, PR2 and RS2 than those samples are to any other, despite their production and life-histories being significantly different from one another. Rows and columns in the heat-map are clustered using correlation distance and average linkage. Rows are centred and unit variance scaling is applied to rows. Despite the close clustering of L1 with other tars, when plotting PC1 and PC2, marked variation is observed in the heat map by peaks at 49.5, 53.9, 46.0, 47.6, and 53.2.

To summarize the results, both condensation samples are similar, so we can conclude based on the biomarkers identified by GC–MS analysis that the condensation method gives the most reproducible results. However, the number of samples is currently too small to guarantee this with a high degree of certainty. Further, the condensation results do not fit into either a single or double pot category. This is expected as the process itself is unique. The GC–MS results of tars made in pit rolls and raised structures are less reproducible and show the most intra-method variation (Fig. 5). Based on the signal intensity, PR1 has the highest concentration of all compounds and there are overall more compounds present than in PR2. Similarly, there is considerable variation in tar made with raised structures. RS1 contains more compounds than does RS2. This may be the result of the abrupt end to the RS2 experiment (Fig. 3), which prevented the formation of further degradation products. When compared with the ash mound method, which most closely resembles that of a single pot tar production, there is less difference between it and RS1 than there is between RS1 and RS2. Likewise, L1 appears closer to PR2 and RS2 than those are to PR1 and RS1, respectively (Fig. 5a), despite L1 being produced in laboratory conditions and subject to a three year natural weathering experiment. The presence or absence of fatty acids in tars made by different methods also shows considerable inter- and intra-method variation. All samples can be accurately identified as birch tar, due to the high concentration of standard biomarkers such as betulin and lupeol, as well as other biomarkers α-betulin I, Lupadiene, and lupadienol. However, in order to securely distinguish Palaeolithic production processes, a much larger sample would need to be provided to account for variation within each single method.

## Discussion

The ability to accurately identify Palaeolithic tar production processes is necessary to further the discussion on Neanderthal adhesive technologies, and through this, their behaviour and cognitive capabilities. Currently, no unambiguous physical remains of Palaeolithic tar production exist in the archaeological record, with the exception of the tar itself. Determining production processes based on specific chemical biomarkers is therefore the most promising way to illuminate ancient production processes.

Previous experiments have shown how both experimental and archaeological production processes utilizing ceramic vessels can be distinguished based on the presence or absence of specific biomarkers. Notably, behenic acid and other saturated fatty acids were shown to be absent from single pot tar, and present in double pot tar. However, these results do not align with our experimental Palaeolithic production processes, nor with the laboratory tar that we produced and analysed using the same GC–MS protocols. It has also been reported that behenic acid was not useful for identifying the production process used to make the Königsaue tar^30^. Behenic acid was found to be absent in both the archaeological samples, and the two experimental samples made as references in their study^30^, yet it is present in all but the ash mound tars made for this study. The use of ceramic vessels in tar production may provide more stable and consistent environments limiting compositional variation. For example, the raised structure creates the most stable internal temperatures of any aceramic technique, yet the oxygen content may fluctuate significantly inside the structure. As the raised structure is heated, the mud dome dries and shrinks, creating small cracks and allowing variable amounts of oxygen in to react with the bark. Cracking is much less likely to occur with pre-fired ceramics.

The absence of betulone from RS2 and PR2, for example, may be due to differences in temperature, as well as oxygen exposure. RS1 and PR1, both of which contain betulone, were heated to a higher maximum temperature and for a longer time than RS2 and PR2. The absence of betulone in L1, which was heated in an open air environment and subject to three years of natural weathering is puzzling, because both of these processes should lead to the formation of more betulone^48^. However, betulone was not identified in the Palaeolithic tar from Königsaue (Germany)^14^, but was found in the Palaeolithic tar from Campitello Quarry (Italy)^13^. It is therefore likely that specific burial conditions play an important role in the further degradation of compounds. In addition, the formation of allobetulane related biomarkers from betulin and betulinic acid catalysed by clay minerals^49^ suggests that the sediment type used during the production of tar may also effect the formation of certain compounds.

Our results also highlight the problem of equifinality in identifying ancient tar production processes, particularly when using PCA. Laboratory-made tar that was subject to open-air heating and natural decay clusters closely with RS2, PR2, and AM1. These tars were produced by entirely different processes and had very different life histories. Despite the high variation within raised structure and pit roll tars, there appears to be a pattern along PC1 wherein tars exposed to more oxygen during production have lower values and tars exposed to less oxygen during production have higher values (Fig. 5a). More experiments will be needed to confirm this. L1, which was heated after production, has PC1 values similar to RS2. Open air heating has been recorded as having little effect on the molecular signatures of both single and double-pot tars^50^, which is supported by our results here. More experiments measuring the thermal decay at different temperatures, as well as other biological and chemical decay is clearly necessary. In a similar vein, the condensation tar may appear unique when initially produced, but it is possible that re-heating and natural decay make these differences more nuanced and harder to recognize.

This pilot study has shown that using GC–MS to identify Palaeolithic birch tar production techniques is clearly fraught with challenges. However, we believe that with well-formulated experiments and the open sharing of data, the task is not impossible. Determining how different biomarkers and degradation products are formed in specific production and burial environments will be an important first step. Larger experimental datasets may help to illuminate patterns in production methods, but the problem of a small archaeological dataset will remain. Experiments should therefore aim to reconstruct details of the adhesive production relevant to specific archaeological sites. Testing the biomarkers of tar produced in different soil types, and their degradation in different pH levels (c.f.^49,51^), as well as measuring the atmospheric composition and oxygen content during production may all help to explain the variation found in aceramic tars. Focusing on individual compounds, such as betulone or behenic acid and testing their responses to different temperature/redox conditions is another approach that may help explain the variation observed here. There may also be compounds, which can help differentiate aceramic production methods that are different from those suggested for ceramic techniques. For example, the presence of certain triterpenoid esters may indicate specific heating conditions^52^. A closer look at GC–MS datasets, exploring the formation of different compounds may therefore reveal patterns that were missed in this study.

## Conclusion

Current methods of distinguishing ceramic tar production processes using GC–MS cannot be reliably used to identify Palaeolithic aceramic tar production techniques. Palaeolithic tar produced by methods which most closely resemble single pot tar production contain key biomarkers that are used to exclude single pot tar production in Neolithic samples^37^. Further, the likelihood that tar samples were subject to open air heating during application or re-use, and thousands of years of natural decay make it difficult to attribute meaning to the absence of key markers. In order to resolve these problems, a more detailed comparison of many experimental samples from different tar production processes is required. Only with a much larger dataset of experimental references, including tars made in different ways, and subject to different degrees of heating and degradation, may we begin to distinguish Palaeolithic tar production processes. For this reason we have also made our data publicly available so that it can be used in detailed comparisons with future research^44^. A large and public dataset will help shed new light on ancient birch tar production strategies. By proxy, we will better understand the technological and cognitive capabilities of Neanderthals, as well as how the oldest adhesive technology was discovered, maintained, and how it evolved through time.

## Data Availability

The data that support the findings of this study are openly available in the 4TU.ResearchData repository: “Gas Chromatography–Mass Spectrometry Data used for "Identifying Palaeolithic birch tar production techniques: challenges from an experimental biomolecular approach”. 4TU.ResearchData. Dataset https://doi.org/10.4121/21908796.
